# The association of lipid metabolism and sarcopenia among older patients: a cross-sectional study

**DOI:** 10.1038/s41598-023-44704-4

**Published:** 2023-10-16

**Authors:** Yiwen Jiang, Bingqing Xu, Kaiyu Zhang, Wenyu Zhu, Xiaoyi Lian, Yihui Xu, Zhe Chen, Lei Liu, Zhengli Guo

**Affiliations:** 1 Peking Union Medical College and Chinese Academy of Medical Sciences, Beijing, 100006 China; 2 Department of Gerontology, Affiliated Kunshan Hospital of Jiangsu University, 566 Qiannjin East Road, Kunshan, Suzhou, 215300 Jiangsu China; 3 Laboratory of Cough, Affiliated Kunshan Hospital of Jiangsu University, Kunshan, 215000 Jiangsu China

**Keywords:** Geriatrics, Dyslipidaemias

## Abstract

Sarcopenia has become a heavy disease burden among the elderly. Lipid metabolism was reported to be involved in many degenerative diseases. This study aims to investigate the association between dysregulated lipid metabolism and sarcopenia in geriatric inpatients. This cross-sectional study included 303 patients aged ≥ 60, of which 151 were diagnosed with sarcopenia. The level of total cholesterol (TC), triglyceride (TG), high-density lipoprotein (HDL), low-density lipoprotein (LDL), homocysteine (HCY), BMI, and fat percentage, were compared between sarcopenia and non-sarcopenia patients. The Spearman correlation coefficient was used to estimate the association between sarcopenia and the level of lipid metabolism. To determine risk factors related to sarcopenia, a multivariate logistic regression analysis was carried out. Risk prediction models were constructed based on all possible data through principal component analysis (PCA), Logistic Regression (LR), Support Vector Machine (SVM), k-Nearest Neighbor (KNN), and eXtreme Gradient Boosting (XGboost). We observed rising prevalence of sarcopenia with increasing age, decreasing BMI, and fat percentage (*p* < 0.001, Cochran Armitage test). Multivariate logistic regression analysis revealed sarcopenia’s risk factors, including older age, male sex, lower levels of BMI, TC, and TG, and higher levels of LDL and HCY (*p* < 0.05). The sarcopenia risk prediction model showed the risk prediction value of sarcopenia, with the highest area under the receiver operating curve (AUC) of 0.775. Our study provided thorough insight into the risk factors associated with sarcopenia. It demonstrated that an increase in lipid metabolism-related parameters (BMI, TG, TC), within normal reference ranges, may be protective against sarcopenia. The present study can illuminate the direction and significance of lipid metabolism-related factors in preventing sarcopenia.

## Introduction

With the growing problem of aging, sarcopenia is becoming a bigger public health issue^1^. Sarcopenia was defined as age-related loss of muscle mass, plus low muscle strength, and/or low physical performance by The Asian Working Group for Sarcopenia (AWGS)^2^. A recent systematic review indicates that the overall prevalence of sarcopenia in Chinese community-dwelling older adults aged over 65 years was 17.4%^3^. The link between low muscle strength and adverse health outcomes is long established. Accumulating evidence has demonstrated that strength and functional declines associated with sarcopenia can in turn contribute to disability, frailty, falls, and mortality^4, 5^. Patients with sarcopenia also have a significantly increased risk of comorbidities such as type 2 diabetes, heart failure, and chronic kidney disease^6–9^. Therefore, due to the high prevalence and disease-associated comorbidity and mortality, sarcopenia contributes significantly to the disease burden of the geriatric disease entity and requires more attention.

There is accumulating evidence suggesting that fatty acids and other intermediates in lipid metabolism play a significant role in modulating skeletal muscle mass and function^10^. Sarcopenia features reduced stem cells and terminally differentiated myofibers, replaced with fatty and fibrous tissue in deuteric alterations^11^. Lipids and their derivatives accumulate both within muscle cells and intercellular compartments, further promoting lipo-toxicity and inducing oxidative stress, mitochondrial malfunction, inflammation, and insulin resistance^12^. Whether and how dyslipidemia is involved in the pathogenesis of sarcopenia remains unclear, and previous studies exploring the association between dyslipidemia and sarcopenia obtained inconsistent results^13, 14^. Fundamental studies underpinning the association between lipid and skeletal muscle function need clinical proof.

Blood lipid profile test is routinely used as biomarkers of lipid metabolism, including low-density lipoprotein cholesterol (LDL-C), total cholesterol (TC), triglyceride (TG), and high-density lipoprotein cholesterol (HDL-C)^15^. The correlation between lipid metabolism and sarcopenia has rarely been investigated in a cross-sectional clinical setting. Therefore, we carried out a cross-sectional study to clarify the association of lipid metabolism status with sarcopenia in Chinese geriatric inpatients.

## Methods

### Study participants

324 patients aged ≥60 hospitalized in the Geriatric Medicine Department of Affiliated Kunshan Hospital of Jiangsu University between October 2019 and August 2021 were included in this cross-sectional study. Due to the inconvenience of carrying out dual-energy X-ray (DXA) examination in the community, only hospitalized patients were included. Exclusion criteria included (1) severe heart failure, liver failure, or kidney failure, (2) uncontrolled diabetes, and other endocrinopathies, (3) received anti-anemic therapy or transfusion within 12 months preceding hospitalization, (4) muscular or neuromuscular disorders, (5) dementia or cognitive impairment, (6) immune disease or treated with steroids, (7) serious acute illness such as acute myocardial infarction, cerebral infarction, subarachnoid hemorrhage, and fractures of the lower extremities. The study protocol was approved by the local ethics (Affiliated Kunshan Hospital of Jiangsu University). All patients provided written informed consent before participation. All methods were performed in accordance with the relevant guidelines and regulations.

### Data collection

Baseline characteristics (age, sex, *etc.*) were obtained, and fat percentage, weight, and bone mineral content were measured with a bioelectrical impedance analyzer (Discovery W (S/N 89064); Hologic Inc, Marlborough, USA) when patients were in stable disease condition without severe inflammation.

Peripheral blood and biochemical parameters included hemoglobin, growth hormone, folate, vitamin B12, 25-hydroxyvitamin D (25-OHD), creatine, homocysteine, and insulin-like growth factor-1 (IGF-1). 25-OHD quantitation was carried out by electrochemiluminescence employing a Roche automatic biochemical immunity analyzer (cobas8000). IGF-1 quantification was performed by enzyme-linked immunosorbent assay.

Hypertension, chronic obstructive pulmonary disease (COPD), atherosclerosis (AS), chronic gastritis, diabetes, smoking status, alcohol consumption status, and cognition status were collected during inpatient service. Bone mineral density was evaluated at the femoral neck, and lumbar spine (L2–L4).

### Blood lipid profile test

We select the peripheral blood lipid profile as a reasonable substitute for lipid metabolism. Serum levels of total cholesterol were determined by cholesterol oxidase, HMMPS method (Fujifilm). Triglyceride (TG) was measured by glycerol-3-phosphate oxidase and glycerol blanking method (Fujifilm). HDL and LDL were both determined by the double reagent direct method. The blood lipid profile was quantified by Spectrophotometer (HITACHI LST008AS). The normal ranges of lipid profiles in the laboratory in our hospital are displayed in Additional file 1 eTable 1. Patients we included all have good control of lipid profile, whether through medication or not. The analyses of medication on blood lipid profile were not performed due to the complexity of medication.

### Assessment of sarcopenia

Sarcopenia was diagnosed according to the criteria proposed by AWGS in 2019^2^. All patients completed the examination when they obtained stable condition without severe inflammation. Appendicular skeletal muscle mass (ASM) was measured by DXA scans by a full-time operator, and the results were reported by three specialized medical personnel (reported by Zhou Qing and Jiang Chengfeng, and checked by Director Zhu Yuchun of the Department of Nuclear Medicine). Relative skeletal muscle mass index (RSMI) (ASM/height^2^) is a prerequisite for the diagnosis of sarcopenia^2^. Muscle mass determines muscle strength and function to a certain extent. Decreased muscle mass will lead to decreased physical activity and increased risk of disability in the elderly. Handgrip strength (of the dominant hand) was measured with a CAMRY EH101 electronic hand dynamometer. In the 6-meter walk test, patients with measured usual gait speed < 1.0 m/s were classified as having positive results. In the 5-time chair stand test, patients were asked to rise from a chair (standard height: 43 cm) with arms folded across their chests 5 times, and results with a total time of 12 seconds or more were defined as positive. In the balance test, patients were to stand first with feet aligned side-by-side, then with one heel at the mid-point of the other foot, and finally with feet aligned in a line, heel to toe. Failure to maintain balance in any of the three postures for more than 10 seconds was defined as a positive result.

Sarcopenia was diagnosed as grip strength and/or usual gait speed reduction, and skeletal muscle mass decline. Skeletal muscle mass declined but physical ability retainment was defined as “pre-sarcopenia”. Skeletal muscle mass, usual gait speed, and grip strength reduction was defined as “severe sarcopenia”. Sarcopenia prevalence was explored by sex and age interval. Lipid profile, BMI, and body fat percentage were stratified by quartiles to explore the association with sarcopenia.

### Statistical analyses and risk model construction

Data are demonstrated as mean ± standard deviation or number (%). Student t-test, rank-sum test, and χ^2^-test were performed to evaluate the differences in body composition, disease, and comorbidity-related factors. Cochran–Armitage test was adopted to evaluate the trend between sarcopenia and lipid profile, BMI, or body fat percentage. Spearman’s correlation rank test was performed to evaluate correlations between sarcopenia and lipid metabolism. Logistic regression analyses were implemented to evaluate sarcopenia risk factors. The thresholds of continuous variables were selected according to clinical significance, and they were transformed into categorical variables based on routine cutoff values in the clinical application. The optimal cutoff values for risk factors were calculated by applying receiver operating characteristic (ROC) analyses.

We use Principal Component Analysis (PCA) to select features in case of multicollinearity. Patients were randomly assigned to training and testing groups according to the ratio of 7:3. We then used the selected features to build sarcopenia risk prediction models through Logistic Regression (LR), Support Vector Machine (SVM), k-Nearest Neighbor (KNN), and eXtreme Gradient Boosting (XGboost). The sensitivity, specificity, positive predictive value (PPV), negative predictive value (NPV), and area under the receiver operating curve (AUC), were applied to assess the accuracy of risk prediction models. Two-sided *p* < 0.05 was considered statistically significant.

### Ethics approval and consent to participate

Ethics Committee of Affiliated Kunshan Hospital of Jiangsu University has approved our research.

## Results

### Patient characteristics 

**Figure 1:**
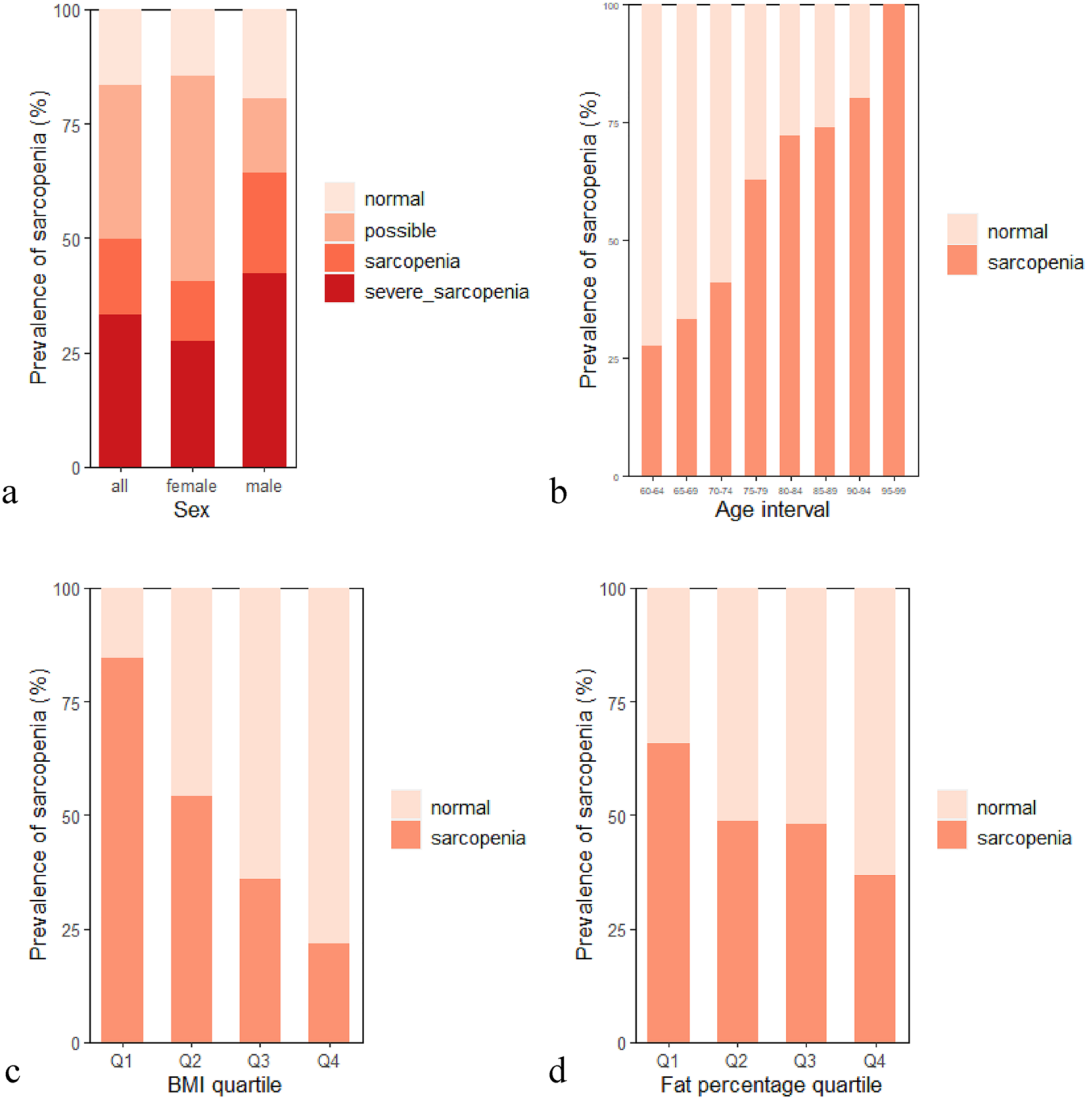
Sarcopenia prevalence (**a**) All patients. (**b**) Assessment of sarcopenia by age. (**c**) Assessment of sarcopenia by BMI quartile. Q1, first quartile, < 20.8 kg/m^2^; Q2, second quartile, 20.8–23.3 kg/m^2^; Q3, third quartile, 23.3–25.8 kg/m^2^; Q4, fourth quartile, > 25.8 kg/m^2^. (**d**) Assessment of sarcopenia by fat percentage quartile. Q1, < 27.7%; Q2, 27.7–33.1%; Q3, 33.1–37.5%; Q4, > 37.5%.

324 geriatric inpatients admitted to the department of Geriatrics from 2019 to 2021 were assessed for eligibility, among which 303 patients were eligible and enrolled. Clinical characteristics of the study population and the statistical difference between the sarcopenia and non-sarcopenia groups are presented in Table 1. Patients’ average age was 73.55 ± 7.66 years; average BMI was 23.33 ± 3.59 kg/m^2^. Sarcopenia was present in 49.8% (151/303) of all patients. 16.5% of patients suffer from pre-sarcopenia, whereas 33.3% suffer from severe sarcopenia (Fig. 1a). The average age of sarcopenia group was higher than non-sarcopenia group (*p* < 0.0001). Weight, fat percentage, and BMI were significantly lower (*p* < 0.05). Sex, growth hormone (GH), folate, vitamin B12, creatine, COPD, and cognition status showed a significant difference between the two groups (*p* < 0.05) (Table 1). Most of the included inpatients were mainly comorbid with cardiovascular and cerebrovascular diseases, hypertension, diabetes, COPD, etc., whose relationships with the prevalence of sarcopenia were analyzed statistically.

**Table 1:** Characteristics of included patients.

Parameter	All	Non-sarcopenia	Sarcopenia	*p*
Height (cm)	158.91 ± 8.51	158.02 ± 8.26	159.82 ± 8.70	0.035*
Weight (kg)	59.00 ± 10.69	62.94 ± 9.97	54.97 ± 9.89	< 0.001***
BMI (kg/m^2^)	23.33 ± 3.59	25.14 ± 3.01	21.47 ± 3.17	< 0.001***
Fat percentage (%)	32.30 ± 6.92	33.98 ± 6.04	30.61 ± 7.35	< 0.001***
Male (N)	118 (39%)	42 (28%)	76 (50%)	< 0.001***
Age (year)	73.55 ± 7.66	70.78 ± 6.74	76.34 ± 7.54	< 0.001***
GH (ng/mL)	0.41 ± 0.65	0.35 ± 0.53	0.48 ± 0.75	0.0174*
Folate (nmol/L)	9.77 ± 4.61	10.16 ± 4.34	9.37 ± 4.84	0.023*
VitB12 (pmol/L)	316.36 ± 154.64	340.74 ± 155.20	291.83 ± 150.64	0.004**
creatine (umol/L)	66.87 ± 20.82	64.73 ± 20.34	69.03 ± 21.14	0.026*
RSMI (kg/m^2^)	5.86 ± 1.09	6.44 ± 0.99	5.28 ± 0.85	< 0.001***
Handgrip strength(kg)	20.43 ± 8.30	22.14 ± 9.19	18.72 ± 6.91	0.002**
6 m gait speed (m/s)	1.01 ± 0.42	1.07 ± 0.43	0.94 ± 0.41	0.013*
Hypertension (N)	179 (59%)	92 (61%)	87 (58%)	0.690
Diabetes (N)	78 (26%)	40 (26%)	38 (25%)	0.922
Osteoporosis (N)	167 (55%)	82 (54%)	85 (56%)	0.768
COPD (N)	57 (19%)	21 (14%)	36 (24%)	0.035*
AS (N)	146 (48%)	79 (52%)	67 (44%)	0.227
Chronic gastritis (N)	70 (23%)	36 (24%)	34 (23%)	0.917
Hospitalisation reason				
Hypertension (N)	114 (38%)	54 (36%)	60 (40%)	0.450
Diabetes (N)	106 (35%)	51 (34%)	55 (36%)	0.600
Osteoporosis (N)	65 (21%)	32 (54%)	33 (56%)	0.865
COPD (N)	18 (6%)	6 (4%)	12 (8%)	0.141
Smoking status (N)				
Never	233 (77%)	120 (79%)	113 (75%)	0.396
Ex-smoker	39 (13%)	17 (11%)	22 (15%)	0.379
Current	31 (10%)	15 (10%)	16 (11%)	0.835
Alcohol consumption (N)				
Never	269 (89%)	135 (89%)	134 (89%)	0.984
Ex-alcoholic	15 (5%)	6 (4%)	9 (6%)	0.419
Current	19 (6%)	11 (7%)	8 (5%)	0.486

* represents p < 0.05; ** represents p < 0.01; *** represents p < 0.001

Sarcopenia prevalence was significantly increased with age (*p* < 0.0001, Cochran–Armitage test; Fig. 1b). In ROC analysis of sarcopenia, best cut-off value of age was 72.5 years (sensitivity: 0.671, specificity: 0.689). For BMI, sarcopenia prevalence in the first quartile was 84.4%, and in the fourth quartile was 22.7%. Sarcopenia prevalence demonstrated a declining trend as BMI increased (*p* < 0.0001; Fig. 1c). Sarcopenia prevalence assessed by fat percentage quartile is presented in Fig. 1d. The first quartile had 65.8% sarcopenia patients, and the fourth quartile had 36.8%. Sarcopenia prevalence showed a declining trend as the fat percentage went up (*p* <0.001; Fig. 1d).

### Comparison of lipid profile between sarcopenia and non-sarcopenia patients

**Figure 2:**
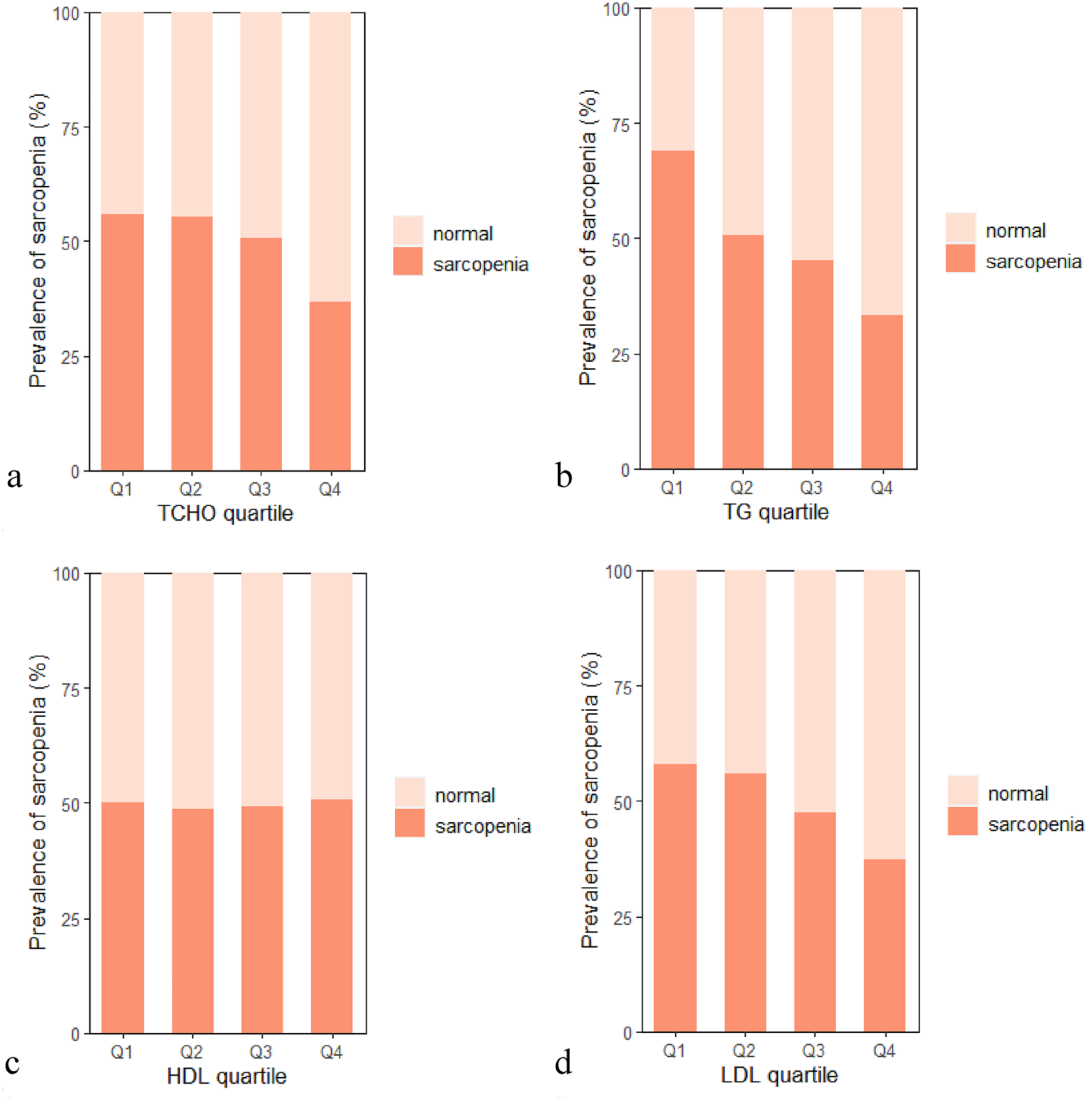
Sarcopenia prevalence (**a**) Assessment of sarcopenia by TC quartile. Q1, < 3.55 mmol/L; Q2, 3.55–4.13 mmol/L; Q3, 4.13–4.81 mmol/L; Q4, > 4.81 mmol/L. (**b**) Assessment of sarcopenia by TG quartile. Q1, < 0.80 mmol/L; Q2, 0.80–1.07 mmol/L; Q3, 1.07–1.55 mmol/L; Q4, > 1.55 mmol/L. (**c**) Assessment of sarcopenia by HDL quartile. Q1, < 1.14 mmol/L; Q2, 1.14–1.33 mmol/L; Q3, 1.33–1.51 mmol/L; Q4, > 1.51 mmol/L. (**d**) Assessment of sarcopenia by LDL quartile. Q1, < 1.97 mmol/L; Q2, 1.97–2.45 mmol/L; Q3, 2.45–3.04 mmol/L; Q4, > 3.04 mmol/L.

We observed significantly different levels of biomarkers in peripheral blood reflecting lipid metabolism among sarcopenic and non-sarcopenia patients (Table 2). Serum levels of TC, TG, and LDL were significantly lower in the sarcopenia group (*p* < 0.01). Homocysteine (HCY) was significantly higher in the sarcopenia group (*p* < 0.01). No significant differences in HDL, 25‐OHD, and IGF-1 were presented between groups. Sarcopenia prevalence showed a significant decrease for increasing lipid profile quartiles (*p* < 0.05, Fig. 2a–d).

**Table 2:** Blood lipid profile test of sarcopenia patiens.

Parameter	Non-sarcopenia	Sarcopenia	*p*
TC (mmol/L)	4.40 ± 1.11	4.07 ± 0.95	0.006**
TG (mmol/L)	1.39 ± 0.71	1.11 ± 0.60	< 0.001***
HDL (mmol/L)	1.35 ± 0.33	1.36 ± 0.34	0.98
LDL (mmol/L)	2.68 ± 0.89	2.39 ± 0.74	0.003**
Height (cm)	158.02 ± 8.26	159.82 ± 8.70	0.035*
Weight (kg)	62.94 ± 9.97	54.97 ± 9.89	< 0.001***
BMI (kg/m^2^)	25.14 ± 3.01	21.47 ± 3.17	< 0.001***
Fat percentage (%)	33.98 ± 6.04	30.61 ± 7.35	< 0.001***
HCY (umol/L)	12.98 ± 4.96	15.60 ± 7.59	0.001**
VitD (ng/mL)	20.38 ± 7.75	20.42 ± 8.51	0.952
IGF-1 (ug/L)	116.26 ± 40.86	115.56 ± 39.33	0.712

* represents p < 0.05; ** represents p < 0.01; *** represents p < 0.001

### Comparison of sarcopenia-related parameters between sarcopenia and non-sarcopenia patients

Table 3 shows sarcopenia-related factors. Sarcopenia group’s RSMI, usual gait speed, and grip strength were lower than non-sarcopenia group (*p* < 0.05). Spearman correlation tests of sarcopenia and lipid metabolism-related parameters show a significant correlation, especially between weight BMI and RSMI (ρ > 0.5, *p* < 0.05) (Additional file 2 eTable 2).

**Table 3:** Sarcopnia-related parameters of non-sarcopenia and sarcopenia patients.

Parameter	All	Non-sarcopenia	Sarcopenia	*p*
RSMI (kg/m2)	5.86 ± 1.09	6.44 ± 0.99	5.28 ± 0.85	< 0.001***
Handgrip strength (kg)	20.43 ± 8.30	22.14 ± 9.19	18.72 ± 6.91	0.002**
Usual gait speed (m/s)	1.01 ± 0.42	1.07 ± 0.43	0.94 ± 0.41	0.013*

* represents p < 0.05; ** represents p < 0.01; *** represents p < 0.001

### Risk factors for sarcopenia

Logistic regression analyses were implemented for sarcopenia risk factors. We took appropriate thresholds of all factors according to their respective clinical standard or best discriminability. Univariate analysis identified age, sex, BMI, fat percentage, TC, TG, LDL, and HCY levels as potential predictors of sarcopenia risk (Table 4). Age, male sex, and HCY level were positively correlated with the probability of incidence sarcopenia. We include these significant factors in the multivariate logistic analysis. Only fat percentage and LDL levels were not significantly related to the probability of incidence sarcopenia. Trends between each significant factor and sarcopenia remain the same.

**Table 4:** Logistic regression analysis for risk factors of sarcopenia patients.

Characteristics	Univariate		Multi-variate	
	OR (95% CI)	*p*	OR (95% CI)	*p*
Age (year)				
≦65	Reference		Reference	
>65	2.81 (1.47–5.4)	0.002**	2.46 (1.20–5.06)	0.014*
Sex				
Female	Reference		Reference	
Male	2.65 (1.65–4.28)	< 0.001***	2.08 (1.11–3.91)	0.023*
BMI (kg/m^2^)				
≦28	Reference		Reference	
> 28	0.20 (0.07–0.55)	0.002**	0.18 (0.06–0.53)	0.002**
Fat percentage (%)				
≦35	Reference		Reference	
> 35	0.53 (0.33–0.85)	0.008**	0.99 (0.53–1.83)	0.968
TC (mmol/L)				
≦0.46	Reference		Reference	
> 0.46	0.46 (0.27–0.76)	0.003**	0.46 (0.22–0.97)	0.042*
TG (mmol/L)				
≦0.41	Reference		Reference	
> 0.41	0.41 (0.25–0.65)	< 0.001***	0.52 (0.30–0.90)	0.019*
HDL (mmol/L)				
≦0.72	Reference			
> 0.72	0.72 (0.39–1.33)	0.295		
LDL (mmol/L)				
≦0.51	Reference		Reference	
> 0.51	0.51 (0.32–0.81)	0.004**	1.23 (0.60–2.52)	0.568
HCY (umol/L)				
≦2.94	Reference		Reference	
> 2.94	2.94 (1.84–4.70)	< 0.001***	2.44 (1.45–4.12)	< 0.001***
VitD (ng/mL)				
≦1.16	Reference			
> 1.16	1.16 (0.73–1.84)	0.527		
IGF-1 (ug/L)				
≦2.07	Reference			
> 2.07	2.07 (0.76–5.67)	0.156		
Atherosclerosis				
No	Reference			
Yes	0.74 (0.47–1.16)	0.186		
Hypertension				
No	Reference			
Yes	0.89 (0.56–1.40)	0.606		

* represents p < 0.05; ** represents p < 0.01; *** represents p < 0.001

### Development and validation of sarcopenia risk prediction model

**Figure 3:**
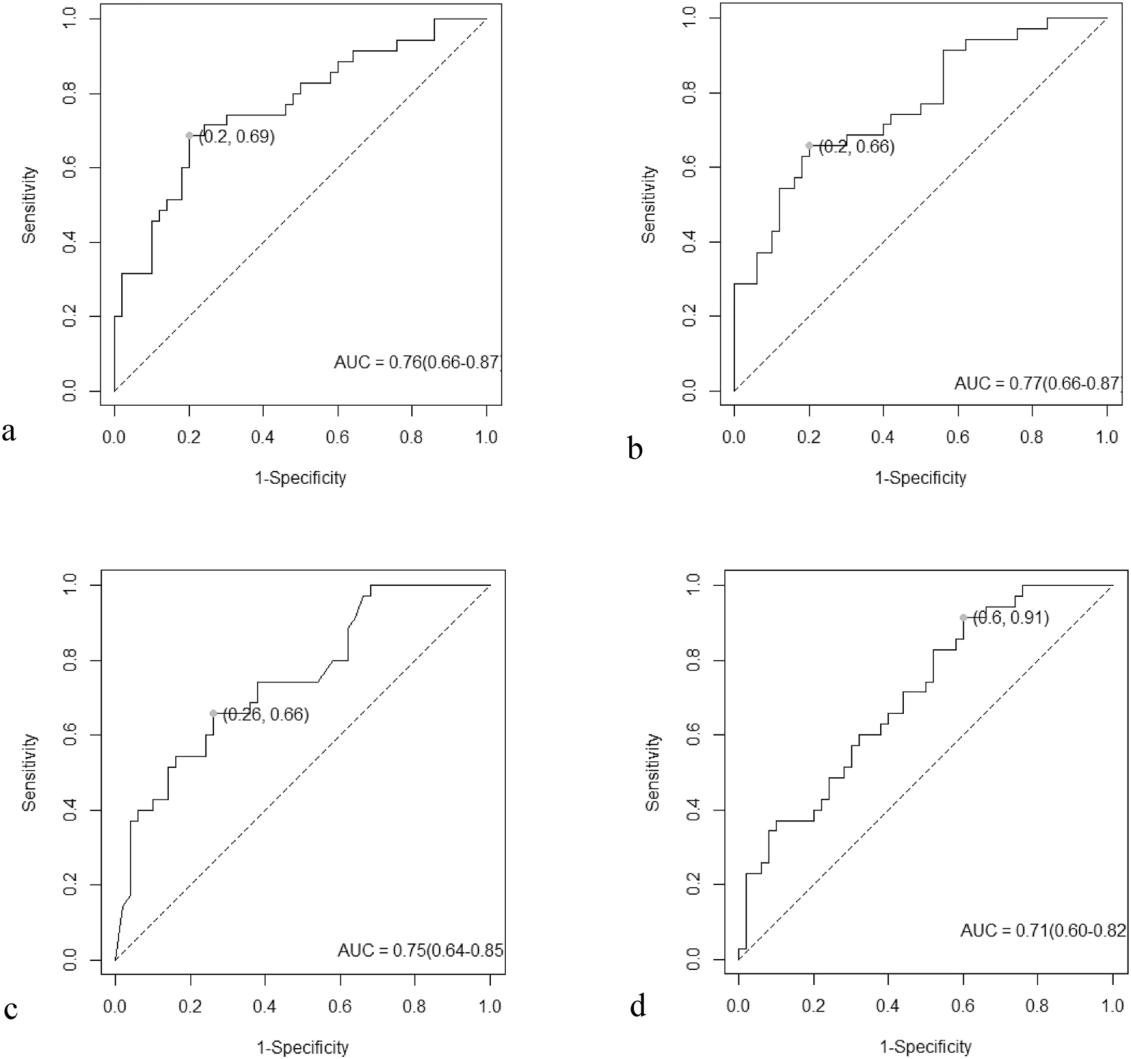
ROC curve of sarcopenia risk prediction model. (**a**) LR; (**b**) SVM; (**c**) KNN; (**d**) XGboost.

All possible data were extracted by PCA as independent features. All patients were randomly assigned into a training group (n = 213) and validation group (n = 85) with 70% and 30% possibility, respectively. LR, SVM, KNN, and XGboost were used to build sarcopenia risk prediction models in training group. Then models’ performance was validated in testing group (Table 5). ROC curves of risk models are demonstrated in Fig. 3. SVM model reaches the highest AUC of 0.775 (Fig. 3).

**Table 5:** Performance of sarcopenia risk models.

Modeling strategy	Sensitivity	Specificity	PPV	NPV	AUC
LR	0.686 (0.532–0.840)	0.800 (0.689–0.911)	0.706 (0.553–0.859)	0.784 (0.671–0.897)	0.764 (0.659–0.869)
SVM	0.657 (0.500–0.814)	0.840 (0.738–0.942)	0.742 (0.588–0.896)	0.778 (0.667–0.889)	0.775 (0.670–0.880)
KNN	0.657 (0.500–0.814)	0.740 (0.618–0.862)	0.639 (0.482–0.796)	0.755 (0.635–0.876)	0.748 (0.643–0.853)
XGboost	0.914 (0.822–1.000)	0.400 (0.264–0.536)	0.516 (0.392–0.641)	0.870 (0.732–1.007)	0.706 (0.596–0.816)

## Discussion

The present research examined the relationship between sarcopenia and lipid metabolism-related parameters. Carried out in geriatric inpatients, this study demonstrated that an increase of lipid metabolism-related parameters (BMI, TG, TC, LDL), within normal reference ranges, may be protective against sarcopenia. We have created a composite multivariate linear model to predict the risk of sarcopenia. An Italian-based study, conducted on 680 elderly participants, presented that within normal ranges, obesity, and metabolic parameters’ level increase, can be protective against muscle loss^16^, which showed the same trend as our findings.

Within normal ranges, higher lipid metabolism-related levels are shown as a protective factor of sarcopenia. During aging, adipose inflammation reallocates fat tissue to the intra-abdominal region, and lipid infiltrates in skeletal muscles, causing physical activity loss^17^. Lipids and their derivatives amass within and around muscle cells, induce mitochondrial malfunction, interfere with fatty acids β-oxidation, and reinforce reactive oxygen species production, causing insulin resistance, lipotoxicity, and enhanced secretion of some pro-inflammatory cytokines^18^. In turn, these cytokines may prompt inflammation, aggravate fatty tissue decrease, and establish a vicious cycle of local hyperlipidemia, inflammation, and insulin resistance that develop systemically, thus intensifying sarcopenic obesity progress^19^.

HCY^20^ was found to be positively correlated with sarcopenia risk. Experimental evidence demonstrates that elevated plasma homocysteine levels may cause toxicity by many mechanisms, including oxidative damage, associated with an increasing aging rate^21^. In human and various animal models of hyperhomocysteinemia (HHCY), a negative correlation has been revealed between HCY and lipoproteins, especially HDL cholesterol (HDL-C)^22^. Researches on HHCY interfering with HDL-C metabolism demonstrated that HCY can reduce circulating HDL-C via inhibiting ApoA-I protein synthesis and enhance HDL-C clearance^23, 24^. The result of our study showed no significant relation between HDL and sarcopenia. HCY may be a substitution for HDL metabolism.

Obesity (assessed by BMI), is associated with lipid metabolism in the elderly. BMI has already been considered a risk factor for many diseases; however, obesity could lead to a better prognosis, called “obesity paradox”^25^. As elucidated by Coutinho^26^ and Hamer and Stamatakis et al.^27^, mortality caused by cardiovascular diseases has demonstrated a negative association with obesity (only BMI-based) in “obesity paradox” circumstances. Our research might be included in “obesity paradox” field. Our study coincides with previous research that presented how obesity mediates malnutrition’s effect on sarcopenia^28^.

This study evaluated the cross-sectional relationships between sarcopenia risk and previous obesity and lipid metabolism-related factors. It helps to understand, how normal ranges of obesity and lipid-metabolic factors may be protective against sarcopenia. This work meets the growing demand to establish and conduct practical methods both at the prevention and management stages for protecting against sarcopenia by lipid metabolism factors. Moreover, the present study can illuminate the direction and significance of lipid metabolism-related factors in preventing sarcopenia. It also demonstrated the potential relevance between lipid metabolism and muscle wasting in the aspect of clinical testing, which has been investigated by several fundamental studies^19^.

Our study has several limitations. Firstly, the issue of causation is impossible to address under a cross-sectional design. Secondly, a larger population is needed to give a solid foundation for the findings and the prediction model.

## Conclusion

In conclusion, our research indicates a clear (cross-sectional) relationship between sarcopenia with lipid metabolism-related factors. More solid evidence supporting our findings could be found by implementing a larger number of participants. Further clinical and experimental studies are needed to validate the influences of interventions to improve lipid metabolism-related sarcopenia.

### Supplementary Information

Supplementary Tables.

## Data Availability

The datasets generated and/or analysed during the current study are not publicly available due to privacy policy but are available from the corresponding author upon reasonable request.
