# Supplementary material for: The association of lipid metabolism and sarcopenia among older patients: a cross-sectional study
Source: Sci Rep. 2023 Oct 16;13:17538. doi: 10.1038/s41598-023-44704-4 (PMC10579328; doi:10.1038/s41598-023-44704-4)
Supplement: Supplementary file 1 — Supplementary Tables. [file 41598_2023_44704_MOESM1_ESM.docx]

**Additional file**

**eTable 1.** Normal ranges of lipid profiles

| **Lipid profile item** | **Normal range** |
| --- | --- |
| **TG** | 0.56-1.47 mmol/L |
| **TCHO** | 3.10-5.69 mmol/L |
| **HDL** | 0.91-1.96 mmol/L |
| **LDL** | 1.67-3.36 mmol/L |
| **HCY** | 0.00-20.0 umol/L |

**eTable 2.** Spearman correlation of sarcopenia and lipid metabolism-related parameters. Asterisk means p < 0.05.

| Parameter | Gait speed | 5 sitstand | | Handgrip strength | RSMI | Balance ability | | | |
| --- | --- | --- | --- | --- | --- | --- | --- | --- | --- |
| Female |  | |  |  |  | | |  | |
| TC | 0.093 | -0.036 | | 0.117 | 0.119 | 0.007 | | | |
| TG | 0.004 | 0.031 | | 0.034 | 0.257* | 0.092 | | | |
| HDL | 0.087 | -0.058 | | 0.127 | -0.074 | -0.130 | | | |
| LDL | 0.079 | -0.002 | | 0.074 | 0.133 | 0.027 | | | |
| Height | 0.199* | -0.147* | | 0.309* | 0.025 | 0.024 | | | |
| weight | 0.113 | -0.086 | | 0.266* | 0.558* | -0.078 | | | |
| BMI | -0.004 | -0.024 | | 0.127 | 0.649* | -0.109 | | | |
| Fat percentage | -0.021 | 0.103 | | 0.040 | 0.023 | 0.064 | | | |
| HCY | -0.129 | 0.164* | | -0.108 | -0.110 | 0.145 | | | |
| VitD | -0.048 | 0.043 | | 0.117 | 0.075 | -0.070 | | | |
| IGF-1 | -0.045 | -0.035 | | 0.090 | -0.023 | -0.094 | | | |
| Male |  | |  |  |  | | |  | |
| TC | 0.159 | -0.213* | | 0.189* | -0.030 | | -0.146 | |  |
| TG | 0.105 | -0.032 | | 0.210* | 0.353* | | -0.116 | |  |
| HDL | 0.038 | -0.202* | | 0.069 | -0.220* | | -0.032 | |  |
| LDL | 0.123 | -0.185* | | 0.229* | 0.058 | | -0.153 | |  |
| Height | 0.086 | -0.026 | | 0.125 | 0.014 | | 0.110 | |  |
| Weight | 0.146 | -0.012 | | 0.426* | 0.713* | | 0.048 | |  |
| BMI | 0.116 | -0.002 | | 0.396* | 0.773* | | -0.026 | |  |
| Fat percentage | -0.052 | 0.190* | | 0.089 | 0.184* | | 0.041 | |  |
| HCY | -0.141 | 0.111 | | -0.170 | -0.146 | | 0.015 | |  |
| VitD | 0.122 | -0.180 | | 0.233* | 0.194* | | -0.136 | |  |
| IGF-1 | 0.094 | 0.039 | | 0.163 | 0.172 | | -0.035 | |  |
